# Genetic similarity and diversity among three camel populations reared in Egypt

**DOI:** 10.1186/s43141-022-00435-z

**Published:** 2022-11-03

**Authors:** Sekena H. Abdel-Aziem, Dalia M. Mabrouk, Heba A. Abd El-Kader, Sally S. Alam, Othman E. Othman

**Affiliations:** Cell Biology Department, National Research Centre, Giza, Egypt

**Keywords:** Camels, mtDNA, Genetic diversity, SNPs, Phylogenetic analysis, 16S

## Abstract

**Background:**

Molecular genetics has been extremely useful in determining the relation between animal populations and documenting the degrees of genetic variation found within them. The present study was undertaken to evaluate genetic diversity and the relationships between the three camel populations reared in Egypt: Maghrabi, Sudani, and Baladi using mitochondrial 16S sequences and other breeds of camels in the world.

**Methods:**

Blood samples were collected from camels belonging to these three populations. Genomic DNA was extracted from the collected blood samples and subjected to PCR using specific primers for mitochondrial 16S region. The amplified products were purified using DNA purification kit to remove residual primers and dNTPs. Sequencing was performed in the Macrogen Incorporation. The amplified products were submitted to GenBank/NCBI under accession numbers OM 278349 and OM 278350

**Results:**

Sequencing was done on the partial mitochondrial 16S amplified fragments at 530 bp. This amplified area had two haplotypes. There was one substitution (G/A) at nucleotide 309 of the amplified segment. The nucleotide (π) and Hd stand for haplotype diversity, respectively, at 0.00008 and 0.042, and the average number of pairwise nucleotide differences, k, is 0.042, according to Fu’s Fs statistic and Tajima’s D, which is −1.10686. Genetic distance percentages between the three populations under study range from 0.000 to 0.0312. A phylogenetic analysis of Egyptian camel populations and other *Camelus dromedarius* populations revealed a strong relationship between them.

**Conclusions:**

This study suggests that the 16S rRNA sequencing in mitochondria plays a critical role in genetic variation studies and analysis of phylogeny between camel populations and breeds.

## Background

Camel is a valuable livestock animal particularly in dry and semidry regions which extend in many African countries [25]. The production yields of camels including meat, milk, and hair as well as their utilization in drought and transportation constitute one of the human needs in large sectors of pastoral societies [27]. There are seven species of camelids are present all over the world belonging to domestic (*Camelus dromedarius*, *Bactrianus*, *Lama glama*, *Vicugna pacos*) and wild (*Camelus dromedarius*, *Bactrianus*, *Lama glama* (*Camelus ferus*, *Lama guanicoe*, *Vicugna vicugna*). Around 37 million camelids are kept globally [12], with the vast majority (around 75%) being. Dromedary and Bactrian camelids are found in the Afro-Asian dryland, the former in Somalia and the latter in Mongolia/China. Camels of the New World (*lama*, *alpaca*, *guanaco*, and *vicuna*) are found mostly in Peru, Chile, and Argentina and have their natural home in the Andean highlands.

In Egypt, the number of camels in 2019 was about 119,885 belonging to five populations: Maghrebi, Somali, Sudani, Baladi (Fallahi), and Mowaled. At the molecular level, three populations (Baladi, Maghrebi, and Sudani) were found to have a high degree of genetic purity with a lower degree of mixing, so they are considered pure populations [4]. These populations are used for different purposes [28], and about half of the Egyptian camels are present in the Shalateen area [20]. The Egyptian camels produce about 20.8, 2.3, 0.62, and 0.09 thousand tons of milk, meat, hides, and fibers, respectively [26]. Camel meat and milk have several advantages over other livestock products, such as lower cholesterol and fat content, and camel milk is more ideal for individuals with allergies than bovine milk [5].

The mitochondria are essential organelles that perform oxidative phosphorylation to produce adenosine triphosphate (ATP), as well as other functions in metabolism, cell signalling, cell cycle regulation, cell differentiation, proliferation, and apoptosis [22]. Because of its small size and the absence of recombination and repair mechanisms, the mitogenome is a circular double-stranded DNA molecule with a high copy rate compared to the nuclear genome and a maternal inheritance pattern that prevents recombination. As a result, the mitogenome sequence is usually stable through generations [14]. Mitochondria have their own DNA (mitogenome), which is considered a powerful resource for evolutionary investigations [6, 34].

Camel ecotype is not well defined in Egypt, and very little information is available about its genetic background. Hence, this study was conducted to find out phylogenetic connection and genetic variation among three camel populations reared in Egypt (Baladi, Sudani, and Maghrabi) using partial mitochondrial 16S region.

The goal of this study is to identify the genetic conservation between three camel’s breeds reared in Egypt using mtDNA *16 s gene* and phylogenic relationships between our tested camels and other breeds of camels in the world to be available for selection of superior animals for breeding.

## Methods

Blood samples of Maghrabi camel were kindly supplied by King Mariout Research Station. While blood samples of Sudani and Falahi (Baladi) were collected from camel market in Berkash, Giza, Egypt. Blood samples were collected from healthy males and females of the three camel breeds. These samples were drawn from the jugular vein of camels into sterile interior plain glass tubes after the addition of EDTA disodium salt (EDTA-Na2) as anticoagulant in each case. Blood samples were used to isolate genomic DNA using phenol/chloroform procedure previously described by Wajid et al. [29]. The extracted DNA was quantified using the NanoDrop spectrophotometer (Thermo Scientific, USA) and stored at −20 °C until further use. A 50 μl reaction mixture including 100 ng genomic DNA, 10× buffer (with 15 mM MgCl2), 10 mM dNTPs mix, 10 pmoles of universal primers [8], and 5 U Taq DNA Polymerase was used to amplify the 16S mitochondrial region.

- 16S F: 5′-GCTATAGAGAAAGTACCGTAAG-3′
- 16S R: 5′-TCATATTAACATTATTGCTTC-3′

The amplification was carried out with initial denaturation at 95 °C for 5 min was followed by 35 cycles of denaturation at 95 °C for 30 s, annealing at 50 °C for 30 s, extension at 72 °C for 45 s, and final extension at 72 °C for 7 min. To eliminate residual primers and dNTPs, the amplified products were purified using a DNA purification kit (ExoSap-IT, USB Corporation) according to the manufacturer’s instructions. The Macrogen Incorporation did the sequencing (Seoul, South Korea).

### Analyze the data

Individual haplotype mutations were identified by aligning mitochondrial 16S sequences from tested camels using the BioEdit software [15]. DnaSP 5.00 software was used to calculate haplotype structure, sequence variation, average number of nucleotide differences (D), and average number of nucleotide substitutions (Dxy) per site comparing breeds [19]. MEGA version 11 software was used to create a neighbor-joining (NJ ) tree for the sequences of tested camel populations as well as a phylogenetic tree between Egyptian camels and other camel breeds across the world [18].

For more illustrative phylogenetic relations, three phylogenetic methods were used in this study: neighbor joining, maximum likelihood, and minimum evolution.

## Results

**Fig. 1:**
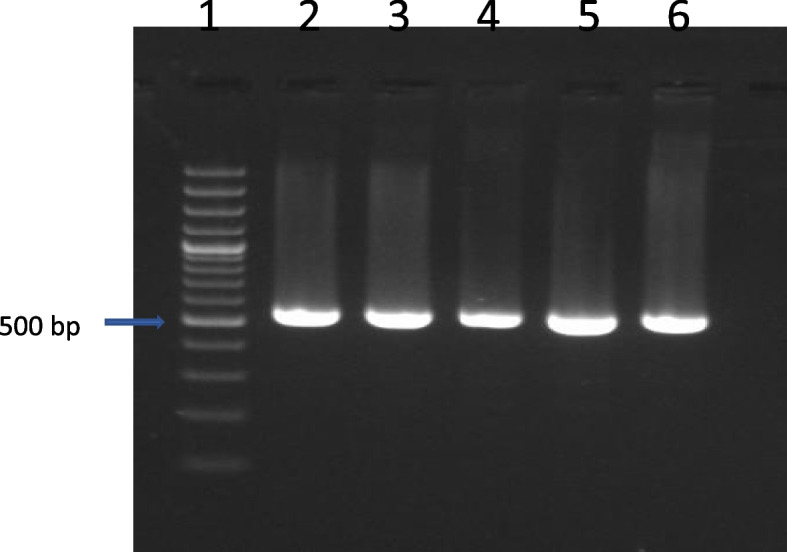
Electrophoresis of PCR products for camel 16S mtDNA. Lane 1, 100-bp DNA molecular marker; lanes 2–6, amplified PCR products at 530 bp

PCR amplified 530-bp fragments from mtDNA 16S mtDNA region of tested camel populations. These amplified fragments were electrophoresed on 1.5% agarose gel (Fig. 1).

**Fig. 2:**
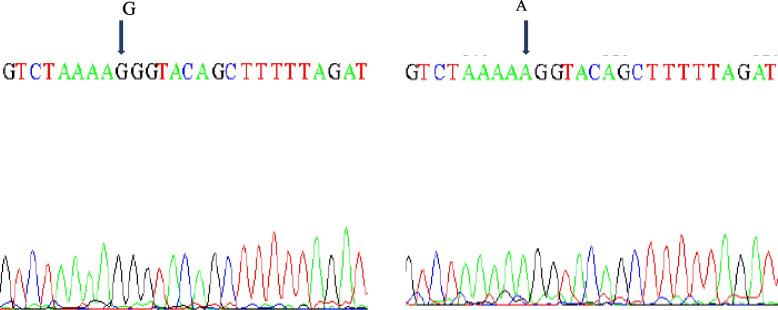
The SNPs G/A at 305 bp

The sequence analysis showed the presence of two haplotypes (forty for haplotype 1 and twenty for haplotype 2) with single-nucleotide polymorphism (SNP) (G/A) at nucleotide no. 309 of 16S amplified region in tested camel populations (Fig. 2).

The nucleotide (π) and haplotype diversities (Hd) in the studied samples were 0.00008 and 0.042, respectively, while the average number of pairwise nucleotide differences (k) was 0.042, Fu’s Fs statistic: −1.602, and Tajima’s D: −1.10686. There were no insertions or deletions observed in any of the 530-bp sequences. The average percentages of the four nitrogen bases were 39.8% (39.7 to 39.9%) for adenine (A), 26.8% (26.6%) for thymine (T), 16.2% (16.4%) for cytosine (C), and 17.2% (17.4 to 17.0%) for guanine (G) (G). In the three camel populations, the mtDNA 16S region displayed a substantial A+T bias (66.6%).

**Fig. 3:**
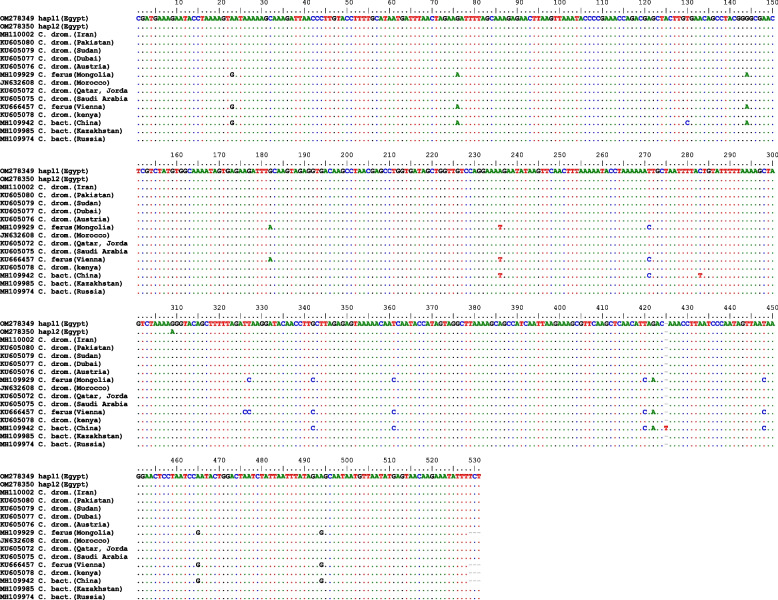
The alignment of partial sequences of the mitochondrial 16S from sixteen camel populations

The two detected haplotypes were submitted to GenBank/NCBI under accession numbers OM 278349 and OM 278350. Alignment of several sequences yielded a consensus length of 531 sites (Fig. 3), which included base pairs and gaps with four InDel sites and InDel haplotype diversity: 0.121. With a transition/transversion bias (R) of 8.99, transitional pairs were more common than transversional pairs on average.

The alignment revealed 100% homology with the sequences obtained from GenBank of camels: KU605080 (Pakistan), KU605079 (Sudan), KU605077 (Dubai), KU605078 (Kenya), KU605076 (Austria), N632608 (Morocco), KU605072 (Qatar, Jordan border), KU605075 (Arabian Peninsula), MH109985 (Kazakhstan), MH109974 (Russia), and MH110002 (Iran). On the other hand, the tested sequences revealed a homology with MH109929 (Mongolia), MH109942 (China), and KU666457 (Vienna) at 97.34%, 97.16%, and 97.15%, respectively.

### Estimating evolutionary distances

Table 1 shows the pairwise genetic distances between the 16 camel populations estimated using the Kimura 2-parameter model. The genetic distances varied between 0.0000 and 0.0312. *C. ferus* (Vienna), and hap 2 of the examined species had the greatest genetic distances (0.0312) in Egyptian camels. There were no genetic distances (0.0000) detected between hap1 (Egypt) and each of camel populations in Iran, Pakistan, Sudan, Austria, Morocco, Dubai, Saudi Arabia, and Kenya and Bactrian camels in Kazakhstan and Russia.

**Table 1:** Genetic distances between 16 camel populations based on mtDNA 16S rRNA gene sequences

	1	2	3	4	5	6	7	8	9	10	11	12	13	14	15	16
**1**																
**2**	**0.0019**															
**3**	**0.0000**	**0.0019**														
**4**	**0.0000**	**0.0019**	**0.0000**													
**5**	**0.0000**	**0.0019**	**0.0000**	**0.0000**												
**6**	**0.0000**	**0.0019**	**0.0000**	**0.0000**	**0.0000**											
**7**	**0.0000**	**0.0019**	**0.0000**	**0.0000**	**0.0000**	**0.0000**										
**8**	**0.0272**	**0.0292**	**0.0272**	**0.0272**	**0.0272**	**0.0272**	**0.0272**									
**9**	**0.0000**	**0.0019**	**0.0000**	**0.0000**	**0.0000**	**0.0000**	**0.0000**	**0.0272**								
**10**	**0.0000**	**0.0019**	**0.0000**	**0.0000**	**0.0000**	**0.0000**	**0.0000**	**0.0272**	**0.0000**							
**11**	**0.0000**	**0.0019**	**0.0000**	**0.0000**	**0.0000**	**0.0000**	**0.0000**	**0.0272**	**0.0000**	**0.0000**						
**12**	**0.0292**	**0.0312**	**0.0292**	**0.0292**	**0.0292**	**0.0292**	**0.0292**	**0.0019**	**0.0292**	**0.0292**	**0.0292**					
**13**	**0.0000**	**0.0019**	**0.0000**	**0.0000**	**0.0000**	**0.0000**	**0.0000**	**0.0272**	**0.0000**	**0.0000**	**0.0000**	**0.0292**				
**14**	**0.0272**	**0.0292**	**0.0272**	**0.0272**	**0.0272**	**0.0272**	**0.0272**	**0.0076**	**0.0272**	**0.0272**	**0.0272**	**0.0096**	**0.0272**			
**15**	**0.0000**	**0.0019**	**0.0000**	**0.0000**	**0.0000**	**0.0000**	**0.0000**	**0.0272**	**0.0000**	**0.0000**	**0.0000**	**0.0292**	**0.0000**	**0.0272**		
**16**	**0.0000**	**0.0019**	**0.0000**	**0.0000**	**0.0000**	**0.0000**	**0.0000**	**0.0272**	**0.0000**	**0.0000**	**0.0000**	**0.0292**	**0.0000**	**0.0272**	**0.0000**	

Lane 1 OM278349 hapl1 (Egypt), lane 2 OM278350 hapl2 (Egypt), lane 3 MH110002 *C. drom.* (Iran), lane 4 KU605080 *C. drom.* (Pakistan), lane 5 KU605079 *C. drom.* (Sudan), lane 6 KU605077 *C. drom.* (Dubai), lane 7 KU605076 *C. drom.* (Austria), lane 8 MH109929 *C. ferus* (Mongolia), lane 9 JN632608 *C. drom.* (Morocco), lane 10 KU605072 *C. drom.* (Qatar, Jordan border), lane 11 KU605075 *C. drom.* (Saudi Arabia), lane 12 KU666457 *C. ferus* (Vienna), lane 13 KU605078 *C. drom.* (Kenya), lane 14 MH109942 *C. bact.* (China), lane 15 MH109985 *C. bact*. (Kazakhstan), lane 16 MH109974 *C. bact.* (Russia)

### Phylogenetic reconstruction

**Fig. 4:**
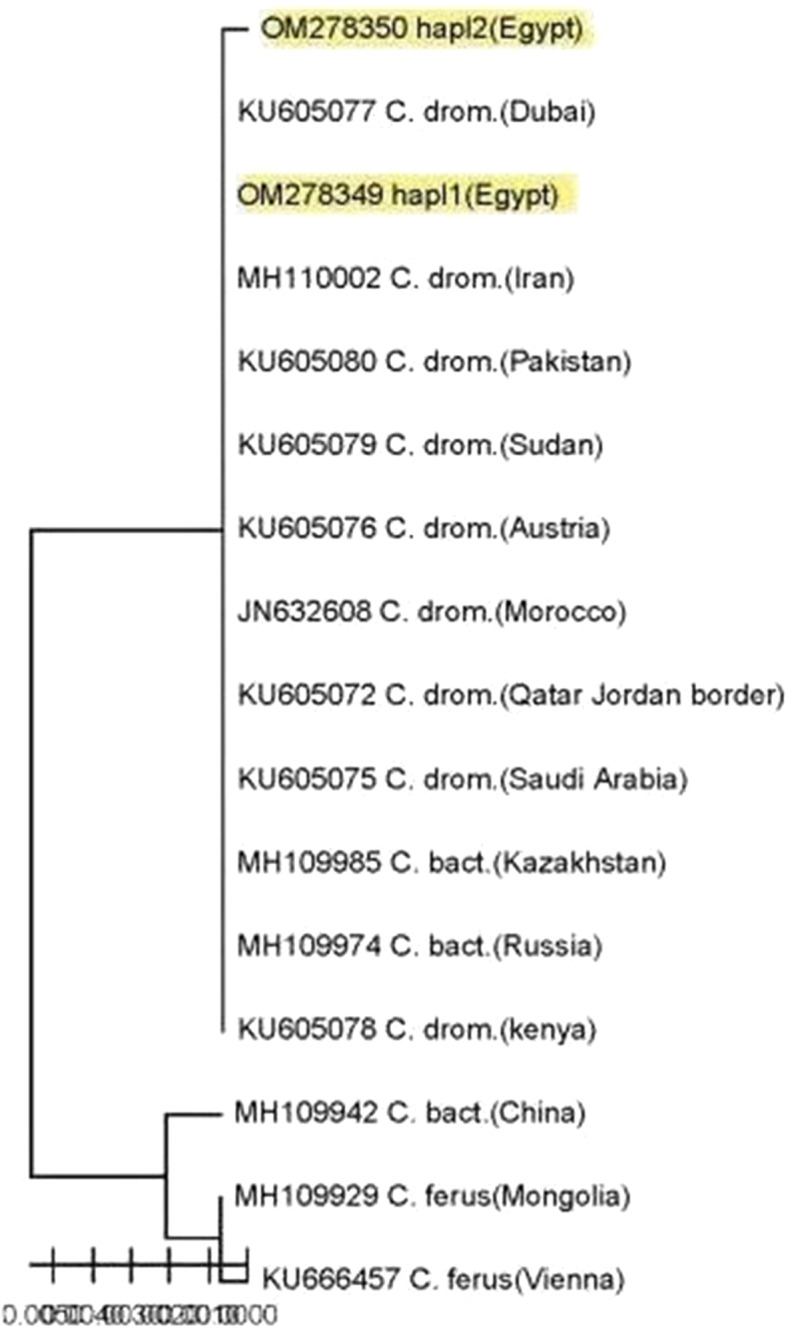
Phylogeny tree constructed by neighbor-joining method among camel populations in Egypt and published sequences using MEGA 11 *computed using the Kimura two-parameter model* using 1000 bootstrap value

**Fig. 5:**
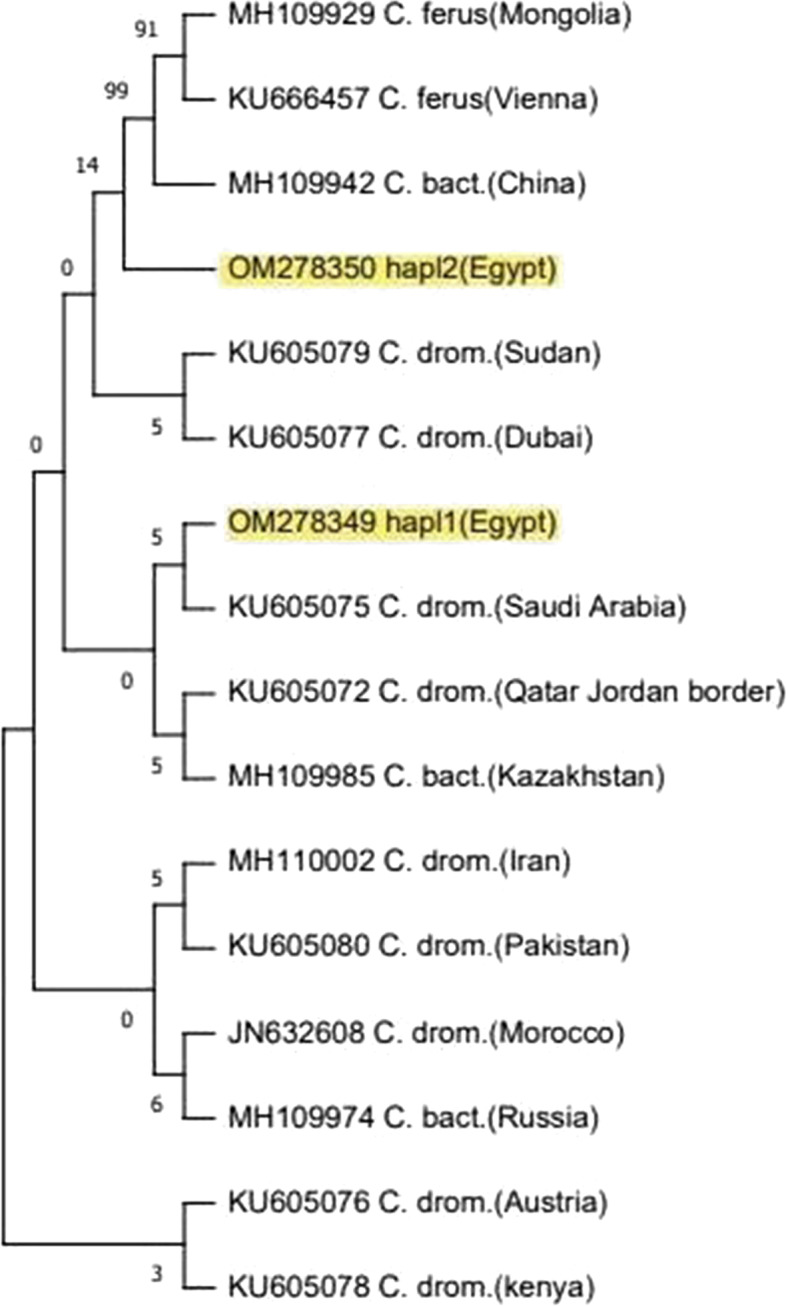
Phylogeny tree constructed by maximum likelihood approach among studied camel populations and other sequences submitted in GenBank/NCBI MEGA 11 *using the Kimura two-parameter model* with 1000 bootstrap value

**Fig. 6:**
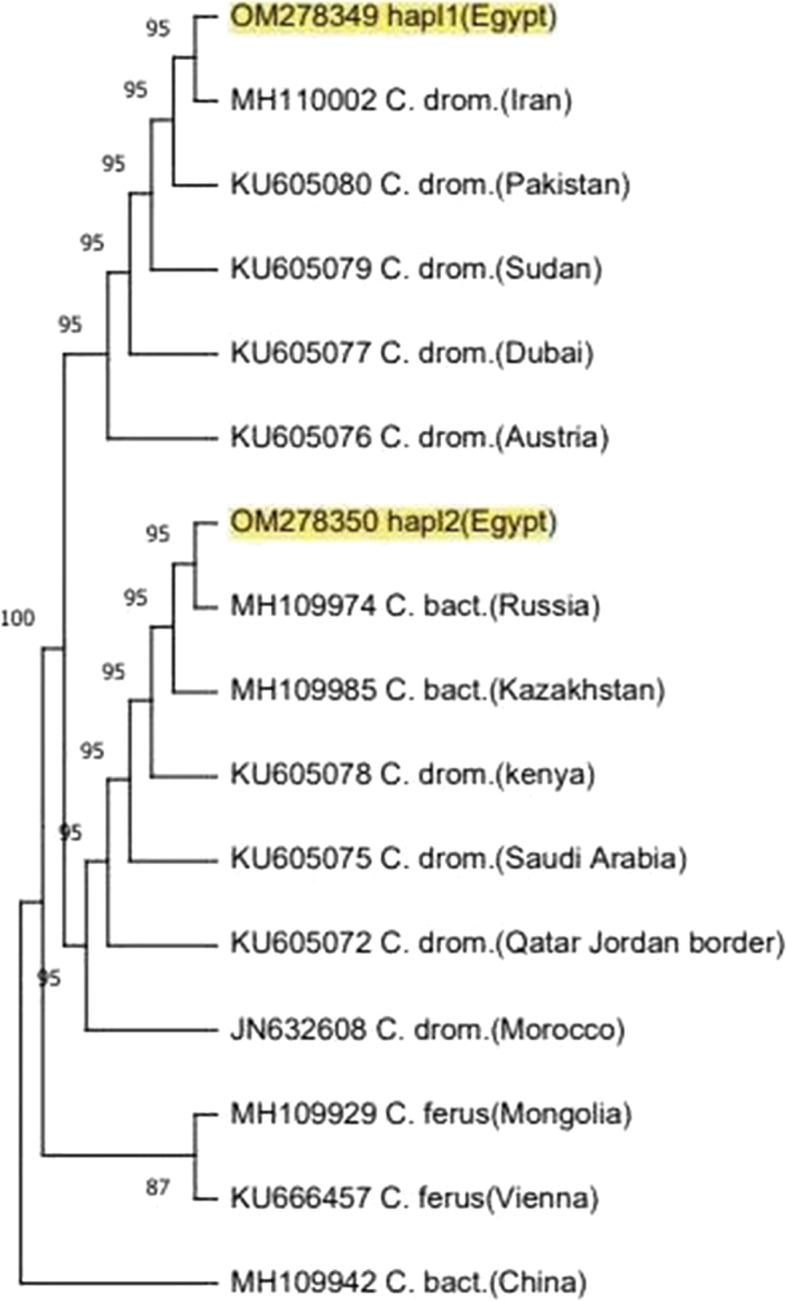
Phylogenetic tree using the minimum evolution (ME) method among camel populations in Egypt and other published sequences using MEGA 11 *computed by Kimura two-parameter model* using 1000 bootstrap value

The phylogeny methods used in the present study showed the same relations among camel populations with some different in support values. All camel haplotypes were separated into two distinct clusters; according to the results, the populations from different geographic regions (Egypt, Sudan, Morocco, Dubai, Qatar, Jordan border, Arabian Peninsula, Pakistan, Iran, Austria, Kazakhstan, and Russia) belonged to one cluster while the second cluster divided into two lineages, the first lineage included ferus populations from Mongolia and Vienna, while the second lineage included Bactrian camel population from China (Figs. 4, 5, and 6).

## Discussion

Mitochondrial DNA (mtDNA) reflects maternal inheritance, and it is valuable for determining genetic variety between animals. It is used to examine genetic variation in dromedaries and to confirm their breeding discrimination and strategies. Between Mongolian domestic and wild Bactrian camels, there was a 1.9% nucleotide variation in the mitochondrial regulatory regions (CR) [17, 30]. Also, sequencing analysis of mtDNA revealed cross-continental mobility and the dynamics of domestication of the dromedary camel, as well as the domestication processes in dromedaries [3].

The present genetic data of *C. dromedarius* compared with other camelid species showed that the modest differences in length were primarily due to differences in the control region. This discovery explains why the regulatory region was first employed to explore genetic variation in mammals due to its quick evolutionary pace compared to protein-coding genes and rRNA [32].

The results revealed the presence of two haplotypes with low genetic differences, and proven Baladi population is more closely related to Sudani population than the Maghrebi population. This finding agrees with the result of Dowidar et al. [10] who reported that Fallahi (Baladi) breed is more closely related to *Camelus dromedarius* (African breed) than the Maghrebi breed, which separated with *Camelus ferus* in one clade and indicating that they share a common ancestor. Furthermore, the present results agree with finding of El-Seoudy et al. [11] who reported that the Maghrebi was independent from Fallahi breed in single clad. On the other hand, Mahrous et al. [21] indicated that the evolutionary links between five Egyptian camel populations revealed two groupings based on random amplified polymorphic (RAPD) and microsatellite, Baladi and Maghreb from the first group and the second group included Sudani.

Our results revealed that the partial sequences of mtDNA 16S in the three tested populations are rich in AT content with an average of 66.6%. These findings support earlier studies that discovered an imbalance in the A+T base composition of mtDNA [16, 23, 33] and noted AT content was found to be substantially greater than GC content, which is consistent with the base composition of mitochondrial genomes from other mammals.

The results revealed only one nucleotide substitution was identified in the 16S regions of sixty sequences of tested camels, and no deletion or insertion of mutations was found. To determine the degree of genetic difference across and within camel populations, the phylogenetic tree and haplotype network were constructed. The two haplotypes, Hap 1 and Hap 2, were shared with fourteen reference populations with a consensus length of 531 sites.

In this study, the average number of pairwise nucleotide differences, k: 0.042 and Fu’s Fs statistic: −1.602 with D: −1.10686 of Tajima, was discovered. The population expansion was determined using Fu’s Fs and Tajima’s *D*-tests, which differ slightly in their approach. The *D*-test of Tajima [31] is calculated by comparing the allelic frequency of segregating nucleotide locations, and a negative value implies that the number of rare alleles is overrepresented. The Fu Fs test [13] is based on the distribution of alleles or haplotypes, and excessively, negative results can imply an excess of alleles, as expected by recent population increase or genetic hitchhiking.

Our results showed that camel populations are slightly different in terms of haplotype and nucleotide diversity, with differences of 0.042 and 0.00008, respectively. Ming et al. [24], who studied eleven domestic camel varieties in China, found lower values. Mongolia, and Russia, as well as a group of Mongolian camels with two humps and reported that the average of haplotype and nucleotide diversity is between 0.456 and 0.0011 to respectively 0.900 and 0.0032. Additionally, Abdussamad et al. [1] reported higher variability in Nigerian camel (0.751 and 0.002 respectively), and Babar et al. [5] found the same results (0.833 and 0.00187, respectively) in Pakistani camels. Moreover, Othman et al. [26] showed that in haplotype and nucleotide diversity in six Egyptian camel populations, the values were 0.241 and 0.00150, respectively.

Present result has found little differences in dromedary camel populations, as evidenced by very low nucleotide diversity (0.00026) and moderate to high haplotype diversity (0.725). When compared to other animals that are more carefully bred for a specific function, these diversity indices may reflect a significantly lower selection pressure on camels [2, 3]. Moreover, there were no genetic differences between Mongolian Bactrian camel groups according to Chuluunbat et al. [7]. Domestic Bactrian camel populations show little genetic difference thought to be due to two factors: one, the historical legacy of Silk Road trading, which favored gene flow between different populations, and two, directional breeding programs based on the use of populations with excellent production performance and economic benefits but a smaller effective population size.

In the present study, an evolutionary tree based on the mtDNA 16S was constructed for better understanding of genetic variation between and within Egyptian camel populations. The phylogenetic tree explained the closest pairwise genetic distance between the two detected haplotypes and camel populations from other fourteen countries as reference populations. The results documented that there is no pairwise genetic distance between Sudani and Baladi populations where they have the same haplotype and the most genetic distance was found between the Baladi, Sudani, and Maghrebi breeds; it suggests that the Baladi and Sudani breeds are more closely linked and share common origins. This result is supported by the bootstrap value of 1000% which is a benchmark to determine the level of accuracy of the phylogeny tree [9]. These findings are nearly like those of Al-Soudy et al. [4], who found that evolutionary relationships based on SCoT and microsatellite markers showed that Maghrebi was separated in a a single cluster, and the second group included two subdivisions, one containing Sudanese and other containing Fallahi. Furthermore, the highest genetic distance reported between Fallahi and Maghrebi contradicted Mahrous et al. [21]’s finding that the evolutionary relationship between five Egyptian camel populations breeds revealed two groups based on RAPD and microsatellite markers, with Fallahi and Maghrebi in one and Sudani in the other.

## Conclusions

Mitochondrial DNA has evolved into a highly effective tool for species identification and forensic science owing to the large number of copies in each cell and the lack of recombination with paternal mtDNA. Current study’s findings validated the 16S sequence’s relevance and effectiveness in measuring genetic variety among camel populations. This data could be utilized as a starting point for developing additional proposals for genetic enhancement and conservation strategies for Egyptian camel genetic resources.

## Data Availability

The authors declare that the data supporting the findings of this study are available within the article.

## Abbreviations

MtDNA: Mitochondrial DNA
SNP: Single nucleotide polymorphism
bp: Base pair
A: Adenine
G: Guanine
C: Cytosine
T: Thymine
dNTPs: Deoxyribonucleotide triphosphate
Mgcl2: Magnesium chloride
PCGs: Protein coding genes
C. *drom.*: *Camelus dromedarius*
C. *bact.*: *Camelus bactrianus*
rRNA: Ribosomal RNA
tRNA: Transfer RNA
PCR: *Polymerase chain reaction*
RAPD: Random amplified polymorphic DNA
inDel: Insertion and deletion

## References

[1] Abdussamad AM, Charruau P, Kalla DJU, Burger PA (2015). Validating local knowledge on camels: colour phenotypes and genetic variation of dromedaries in the Nigeria-Niger corridor. Livest Sci.

[2] Alaqeely R, Alhajeri BH, Almathen F, Alhaddad H (2021). Mitochondrial sequence variation, haplotype diversity, and relationships among dromedary camel-types. Front Genet.

[3] Almathen F, Charruau P, Mohandesan E, Mwacharo JM, Orozco-terWengel P, Pitt D (2016). Ancient and modern DNA reveal dynamics of domestication and cross-continental dispersal of the dromedary. Proc Natl Acad Sci U S A.

[4] Al-Soudy A, El-Sayed A, El-Itriby H, Hussein E (2018) Assessment of the genetic diversity, breeds structure and genetic relationships in four Egyptian camel breeds using microsatellite and start codon targeted (SCoT) markers. Biodivers Endanger Species. 10.4172/2332-2543.S2-001

[5] Babar ME, Hussain T, Wajid A, Nawaz A, Nadeem A, Shah SA, Abdullah M (2015). Mitochondrial cytochrome-b and D-loop sequence-based genetic diversity in Mareecha and Bareela camel breeds of Pakistan. J Anim Plant Sci.

[6] Chen XJ (2013). Mechanism of homologous recombination and implications for aging-related deletions in mitochondrial DNA. Microbiol Mol Biol Rev.

[7] Chuluunbat B, Charruau P, Silbermayr K, Khorloojav T, Burger PA (2014). Genetic diversity and population structure of Mongolian domestic Bactrian camels (*Camelus bactrianus*). Anim Genet.

[8] Cui P, Ji R, Ding F, Qi D, Gao H, Meng H (2007). A complete mitochondrial genome sequence of the wild two-humped camel (camelus bactrianus ferus): an evolutionary history of camelidae. BMC Genomics.

[9] Dharmayant I (2011). Filogenetika Molekuler: Metode Taksonomi organisme Berdasarkan Sejarah Evolusi. Wartazoa..

[10] Dowidar YA, Zeidan AE, Badr RM, Mohammed WW, Shehab AM (2022). Evolutionary relationship between Arabian camel breeds based on sequencing of heat-stress genes under Egyptian conditions. Int J Vet Sci.

[11] El-Seoudy AA, El-Salam AZ, Tharwat EE, EL-Salam FA. (2008). Molecular genetic identification of some camel breeds in Egypt. Egypt J Genet Cytol.

[12] FAO (2014). Food and Agriculture Organization of the United Nations. Statistics of live animals.

[13] Fu XY (1997). Statistical tests of neutrality of mutations against population growth, hitchhiking, and background selection. Genetics.

[14] Gupta A, Bhardwaj A, Sharma P, Pal Y, Mamta (2015). Mitochondrial DNA- a tool for phylogenetic and biodiversity search in equines. J Biodivers Endanger Species.

[15] Hall TA (1999). Bio Edit: a user-friendly biological sequence alignment editor and analysis program for windows 95/98/ NT. Nucleic Acids Symp Ser.

[16] Hu XD, Gao LZ (2016). The complete mitochondrial genome of domestic sheep, ovis aries. Mitochondrial DNA A DNA Mapp Seq Anal.

[17] Ji R, Cui P, Ding F, Geng J, Gao H, Zhang H (2009). Monophyletic origin of domestic Bactrian camel (Camelus bactrianus) and its evolutionary relationship with the extant wild camel (Camelus bactrianus Ferus). Anim Genet.

[18] Kumar S, Stecher G, Tamura K (2016). MEGA7: Molecular Evolutionary Genetics Analysis version 7.0 for bigger datasets. Mol Biol Evol.

[19] Librado P, Rozas J (2009). DnaSP, DNA polymorphism analyses by the coalescent and other methods. Bioinformatics.

[20] Mahran OM (2004). Some studies on blood parasites in camels (*Camelus dromedarius*) at Shalatin city, Red Sea Governorate. Assiut Vet Med J.

[21] Mahrous KF, Hassan AIR, Sekena HA, Mohamed AM, Dalia MH (2011). Genetic variations between camel breeds using microsatellite markers and RAPD techniques. J Appl Biosci.

[22] Mandal S, Lindgren AG, Srivastava AS, Clark AT, Banerjee U (2011). Mitochondrial function controls proliferation and early differentiation potential of embryonic stem cells. Stem Cells.

[23] Manee MM, Alshehri MA, Binghadir SA, Aldhafer SH, Alswailem RM, Algarni AT, Al-Shomrani BM, Al-Fageeh MB (2019). Comparative analysis of camelid mitochondrial genomes. J Genet.

[24] Ming L, Yi L, Sa R, Wang ZX, Wang Z, Ji R (2016). Genetic diversity and phylogeographic structure of Bactrian camels shown by mitochondrial sequence variations. Anim Genet.

[25] Mirkena T, Walelign E, Tewolde N (2018). Camel production systems in Ethiopia: a review of literature with notes on MERS-CoV risk factors. Pastoralism.

[26] Othman E, Abd El-Kader HA, Alam SS, Abd El-Aziem SH (2017). Cytochrome b conservation between six camel breeds reared in Egypt. JGEB.

[27] Othman O, Nowier A, El-Denary M (2016). Genetic variations in two casein genes among Maghrabi camels reared in Egypt. Biosci Biotechnol Res Asia.

[28] Ramadan S, Inoue-murayama M (2017). Advances in camel genomics and their applications: a review. J Anim Genetic.

[29] Wajid A, Wasim M, Yaqub T, Firyal S, Tayyab M, Siddique S, Anjum A, Babar M, Hussain T (2014). Assessment of genetic diversity in Balochi and Rakhshani sheep breeds of Balochistan using microstllite and markers. J Anim Plant Sci.

[30] Silbermayr K, Orozco-terWengel P, Charruau P, Enkhbileg D, Walzer C, Vogl C, Schwarzenberger F, Kaczensky P, Burger PA (2010). High mitochondrial differentiation levels between wild and domestic Bactrian camels: a basis for rapid detection of maternal hybridization. Anim Genet.

[31] Tajima F (1989). Statistical method for testing the neutral mutation hypothesis by DNA polymorphism. Genetics..

[32] Tang AMC, Jeewon R, Hyde KD (2009). A re-evaluation of the evolutionary relationships within the Xylariaceae based on ribosomal and protein-coding gene sequences. Fungal Divers.

[33] Xiao D, Hu X, Chen Y, Gong Z, Chen L (2016). The complete sequence of mitochondrial genome of Wuyi black pig (*Sus Scrofa*). Mitochondrial DNA Part A.

[34] Zinovkina LA (2018). Mechanisms of mitochondrial DNA repair in mammals. Biochemistry (Mosc).

